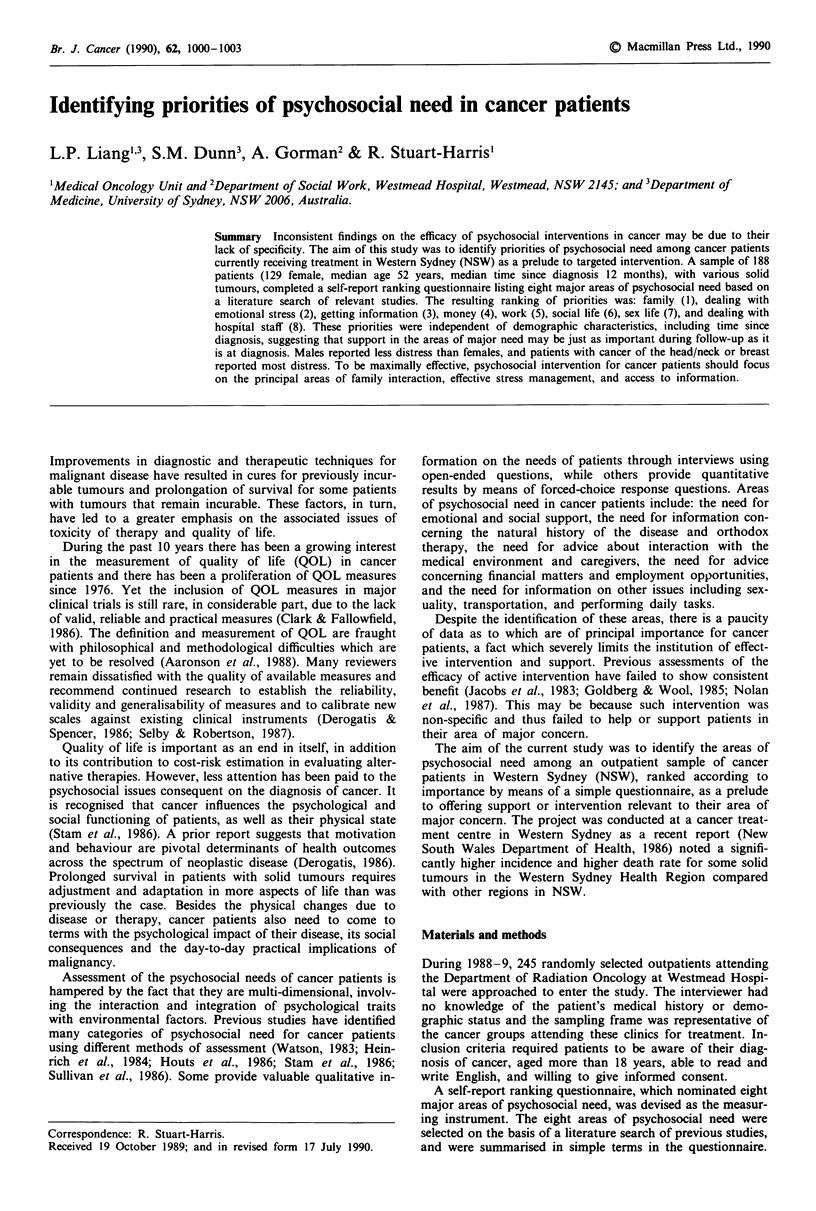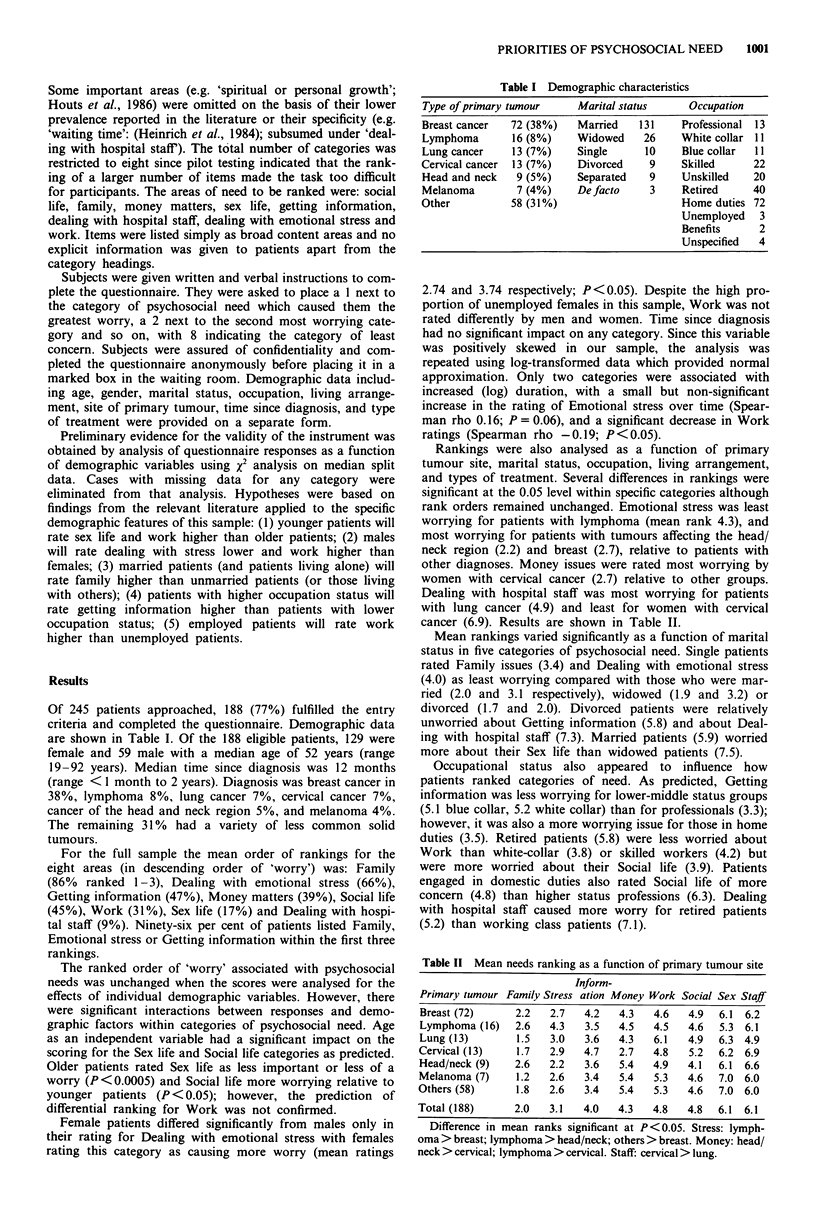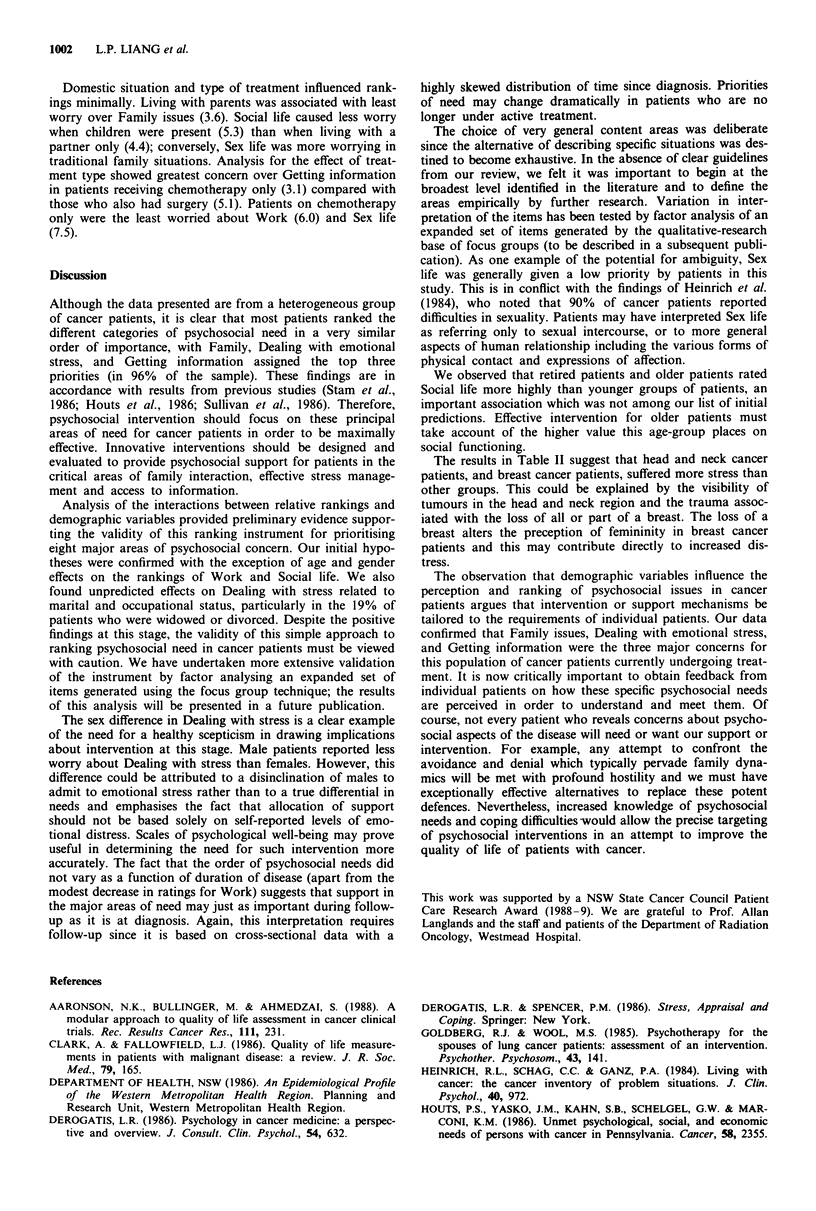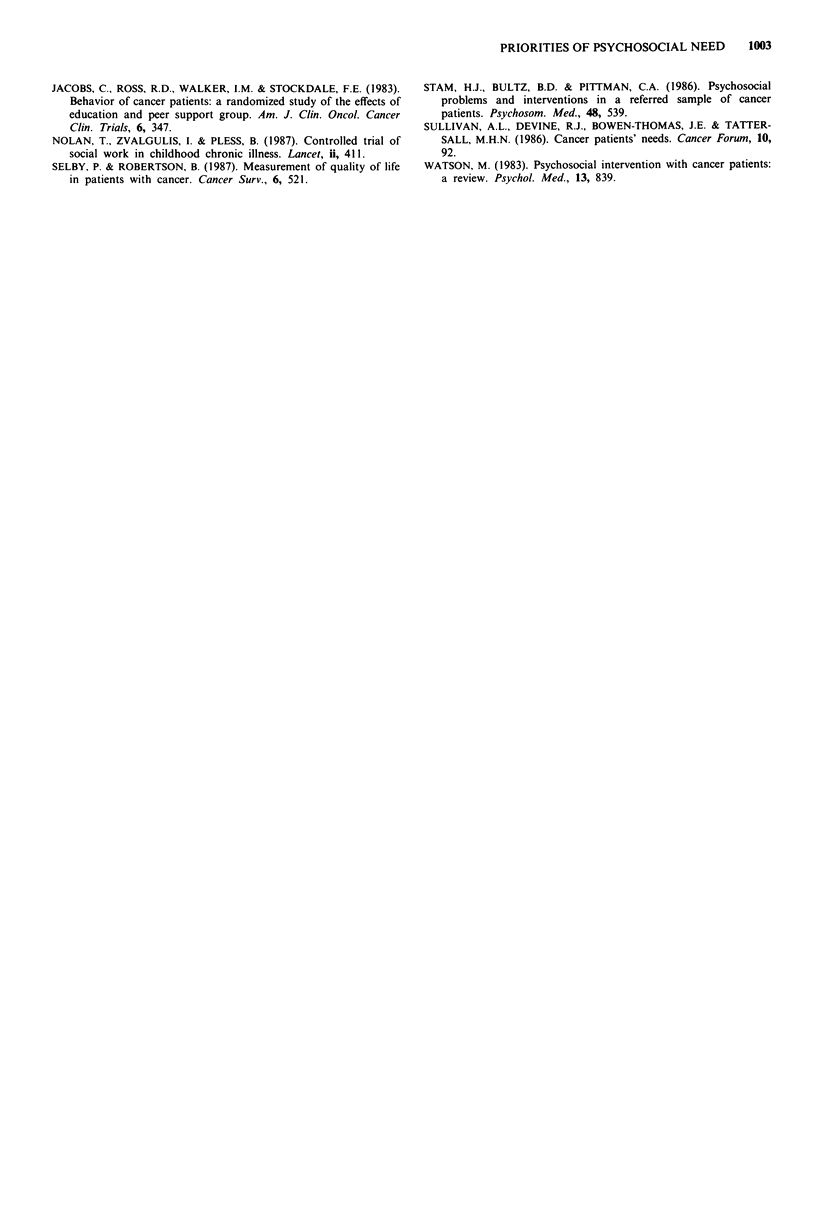# Identifying priorities of psychosocial need in cancer patients.

**DOI:** 10.1038/bjc.1990.425

**Published:** 1990-12

**Authors:** L. P. Liang, S. M. Dunn, A. Gorman, R. Stuart-Harris

**Affiliations:** Medical Oncology Unit, Westmead Hospital, NSW, Australia.

## Abstract

Inconsistent findings on the efficacy of psychosocial interventions in cancer may be due to their lack of specificity. The aim of this study was to identify priorities of psychosocial need among cancer patients currently receiving treatment in Western Sydney (NSW) as a prelude to targeted intervention. A sample of 188 patients (129 female, median age 52 years, median time since diagnosis 12 months), with various solid tumours, completed a self-report ranking questionnaire listing eight major areas of psychosocial need based on a literature search of relevant studies. The resulting ranking of priorities was: family (1), dealing with emotional stress (2), getting information (3), money (4), work (5), social life (6), sex life (7), and dealing with hospital staff (8). These priorities were independent of demographic characteristics, including time since diagnosis, suggesting that support in the areas of major need may be just as important during follow-up as it is at diagnosis. Males reported less distress than females, and patients with cancer of the head/neck or breast reported most distress. To be maximally effective, psychosocial intervention for cancer patients should focus on the principal areas of family interaction, effective stress management, and access to information.


 © Macmillan Press Ltd., 1990

### Identifying priorities of psychosocial need in cancer patients

L.P. Liang[1,3], S.M. Dunn[3], A. Gorman[2] & R. Stuart-Harris[1]

[1]*Medical Oncology Unit and* [2]*Department of Social Work, Westmead Hospital, Westmead, NSW 2145; and* [3]*Department of Medicine, University of Sydney, NSW 2006, Australia.*

**Summary** Inconsistent findings on the efficacy of psychosocial interventions in cancer may be due to their lack of specificity. The aim of this study was to identify priorities of psychosocial need among cancer patients currently receiving treatment in Western Sydney (NSW) as a prelude to targeted intervention. A sample of 188 patients (129 female, median age 52 years, median time since diagnosis 12 months), with various solid tumours, completed a self-report ranking questionnaire listing eight major areas of psychosocial need based on a literature search of relevant studies. The resulting ranking of priorities was: family (1), dealing with emotional stress (2), getting information (3), money (4), work (5), social life (6), sex life (7), and dealing with hospital staff (8). These priorities were independent of demographic characteristics, including time since diagnosis, suggesting that support in the areas of major need may be just as important during follow-up as it is at diagnosis. Males reported less distress than females, and patients with cancer of the head/neck or breast reported most distress. To be maximally effective, psychosocial intervention for cancer patients should focus on the principal areas of family interaction, effective stress management, and access to information.

Improvements in diagnostic and therapeutic techniques for malignant disease have resulted in cures for previously incurable tumours and prolongation of survival for some patients with tumours that remain incurable. These factors, in turn, have led to a greater emphasis on the associated issues of toxicity of therapy and quality of life.

During the past 10 years there has been a growing interest in the measurement of quality of life (QOL) in cancer patients and there has been a proliferation of QOL measures since 1976. Yet the inclusion of QOL measures in major clinical trials is still rare, in considerable part, due to the lack of valid, reliable and practical measures (Clark & Fallowfield, 1986). The definition and measurement of QOL are fraught with philosophical and methodological difficulties which are yet to be resolved (Aaronson *et al.*, 1988). Many reviewers remain dissatisfied with the quality of available measures and recommend continued research to establish the reliability, validity and generalisability of measures and to calibrate new scales against existing clinical instruments (Derogatis & Spencer, 1986; Selby & Robertson, 1987).

Quality of life is important as an end in itself, in addition to its contribution to cost-risk estimation in evaluating alternative therapies. However, less attention has been paid to the psychosocial issues consequent on the diagnosis of cancer. It is recognised that cancer influences the psychological and social functioning of patients, as well as their physical state (Stam *et al.*, 1986). A prior report suggests that motivation and behaviour are pivotal determinants of health outcomes across the spectrum of neoplastic disease (Derogatis, 1986). Prolonged survival in patients with solid tumours requires adjustment and adaptation in more aspects of life than was previously the case. Besides the physical changes due to disease or therapy, cancer patients also need to come to terms with the psychological impact of their disease, its social consequences and the day-to-day practical implications of malignancy.

Assessment of the psychosocial needs of cancer patients is hampered by the fact that they are multi-dimensional, involving the interaction and integration of psychological traits with environmental factors. Previous studies have identified many categories of psychosocial need for cancer patients using different methods of assessment (Watson, 1983; Heinrich *et al.*, 1984; Houts *et al.*, 1986; Stam *et al.*, 1986; Sullivan *et al.*, 1986). Some provide valuable qualitative information on the needs of patients through interviews using open-ended questions, while others provide quantitative results by means of forced-choice response questions. Areas of psychosocial need in cancer patients include: the need for emotional and social support, the need for information concerning the natural history of the disease and orthodox therapy, the need for advice about interaction with the medical environment and caregivers, the need for advice concerning financial matters and employment opportunities, and the need for information on other issues including sexuality, transportation, and performing daily tasks.

Despite the identification of these areas, there is a paucity of data as to which are of principal importance for cancer patients, a fact which severely limits the institution of effective intervention and support. Previous assessments of the efficacy of active intervention have failed to show consistent benefit (Jacobs *et al.*, 1983; Goldberg & Wool, 1985; Nolan *et al.*, 1987). This may be because such intervention was non-specific and thus failed to help or support patients in their area of major concern.

The aim of the current study was to identify the areas of psychosocial need among an outpatient sample of cancer patients in Western Sydney (NSW), ranked according to importance by means of a simple questionnaire, as a prelude to offering support or intervention relevant to their area of major concern. The project was conducted at a cancer treatment centre in Western Sydney as a recent report (New South Wales Department of Health, 1986) noted a significantly higher incidence and higher death rate for some solid tumours in the Western Sydney Health Region compared with other regions in NSW.

### Materials and methods

During 1988-9, 245 randomly selected outpatients attending the Department of Radiation Oncology at Westmead Hospital were approached to enter the study. The interviewer had no knowledge of the patient's medical history or demographic status and the sampling frame was representative of the cancer groups attending these clinics for treatment. Inclusion criteria required patients to be aware of their diagnosis of cancer, aged more than 18 years, able to read and write English, and willing to give informed consent.

A self-report ranking questionnaire, which nominated eight major areas of psychosocial need, was devised as the measuring instrument. The eight areas of psychosocial need were selected on the basis of a literature search of previous studies, and were summarised in simple terms in the questionnaire.

Correspondence: R. Stuart-Harris.
Received 19 October 1989; and in revised form 17 July 1990.

Some important areas (e.g. 'spiritual or personal growth'; Houts *et al.*, 1986) were omitted on the basis of their lower prevalence reported in the literature or their specificity (e.g. 'waiting time': (Heinrich *et al.*, 1984); subsumed under 'dealing with hospital staff'). The total number of categories was restricted to eight since pilot testing indicated that the ranking of a larger number of items made the task too difficult for participants. The areas of need to be ranked were: social life, family, money matters, sex life, getting information, dealing with hospital staff, dealing with emotional stress and work. Items were listed simply as broad content areas and no explicit information was given to patients apart from the category headings.

Subjects were given written and verbal instructions to complete the questionnaire. They were asked to place a 1 next to the category of psychosocial need which caused them the greatest worry, a 2 next to the second most worrying category and so on, with 8 indicating the category of least concern. Subjects were assured of confidentiality and completed the questionnaire anonymously before placing it in a marked box in the waiting room. Demographic data including age, gender, marital status, occupation, living arrangement, site of primary tumour, time since diagnosis, and type of treatment were provided on a separate form.

Preliminary evidence for the validity of the instrument was obtained by analysis of questionnaire responses as a function of demographic variables using $\chi^2$ analysis on median split data. Cases with missing data for any category were eliminated from that analysis. Hypotheses were based on findings from the relevant literature applied to the specific demographic features of this sample: (1) younger patients will rate sex life and work higher than older patients; (2) males will rate dealing with stress lower and work higher than females; (3) married patients (and patients living alone) will rate family higher than unmarried patients (or those living with others); (4) patients with higher occupation status will rate getting information higher than patients with lower occupation status; (5) employed patients will rate work higher than unemployed patients.

### Results

Of 245 patients approached, 188 (77%) fulfilled the entry criteria and completed the questionnaire. Demographic data are shown in Table I. Of the 188 eligible patients, 129 were female and 59 male with a median age of 52 years (range 19–92 years). Median time since diagnosis was 12 months (range < 1 month to 2 years). Diagnosis was breast cancer in 38%, lymphoma 8%, lung cancer 7%, cervical cancer 7%, cancer of the head and neck region 5%, and melanoma 4%. The remaining 31% had a variety of less common solid tumours.

For the full sample the mean order of rankings for the eight areas (in descending order of 'worry') was: Family (86% ranked 1–3), Dealing with emotional stress (66%), Getting information (47%), Money matters (39%), Social life (45%), Work (31%), Sex life (17%) and Dealing with hospital staff (9%). Ninety-six per cent of patients listed Family, Emotional stress or Getting information within the first three rankings.

The ranked order of 'worry' associated with psychosocial needs was unchanged when the scores were analysed for the effects of individual demographic variables. However, there were significant interactions between responses and demographic factors within categories of psychosocial need. Age as an independent variable had a significant impact on the scoring for the Sex life and Social life categories as predicted. Older patients rated Sex life as less important or less of a worry ($P<0.0005$) and Social life more worrying relative to younger patients ($P<0.05$); however, the prediction of differential ranking for Work was not confirmed.

Female patients differed significantly from males only in their rating for Dealing with emotional stress with females rating this category as causing more worry (mean ratings

**Table I** Demographic characteristics

| *Type of primary tumour* | | *Marital status* | | *Occupation* | |
|---|---|---|---|---|---|
| Breast cancer | 72 (38%) | Married | 131 | Professional | 13 |
| Lymphoma | 16 (8%) | Widowed | 26 | White collar | 11 |
| Lung cancer | 13 (7%) | Single | 10 | Blue collar | 11 |
| Cervical cancer | 13 (7%) | Divorced | 9 | Skilled | 22 |
| Head and neck | 9 (5%) | Separated | 9 | Unskilled | 20 |
| Melanoma | 7 (4%) | *De facto* | 3 | Retired | 40 |
| Other | 58 (31%) | | | Home duties | 72 |
| | | | | Unemployed | 3 |
| | | | | Benefits | 2 |
| | | | | Unspecified | 4 |

2.74 and 3.74 respectively; $P<0.05$). Despite the high proportion of unemployed females in this sample, Work was not rated differently by men and women. Time since diagnosis had no significant impact on any category. Since this variable was positively skewed in our sample, the analysis was repeated using log-transformed data which provided normal approximation. Only two categories were associated with increased (log) duration, with a small but non-significant increase in the rating of Emotional stress over time (Spearman rho 0.16; $P=0.06$), and a significant decrease in Work ratings (Spearman rho $-0.19$; $P<0.05$).

Rankings were also analysed as a function of primary tumour site, marital status, occupation, living arrangement, and types of treatment. Several differences in rankings were significant at the 0.05 level within specific categories although rank orders remained unchanged. Emotional stress was least worrying for patients with lymphoma (mean rank 4.3), and most worrying for patients with tumours affecting the head/neck region (2.2) and breast (2.7), relative to patients with other diagnoses. Money issues were rated most worrying by women with cervical cancer (2.7) relative to other groups. Dealing with hospital staff was most worrying for patients with lung cancer (4.9) and least for women with cervical cancer (6.9). Results are shown in Table II.

Mean rankings varied significantly as a function of marital status in five categories of psychosocial need. Single patients rated Family issues (3.4) and Dealing with emotional stress (4.0) as least worrying compared with those who were married (2.0 and 3.1 respectively), widowed (1.9 and 3.2) or divorced (1.7 and 2.0). Divorced patients were relatively unworried about Getting information (5.8) and about Dealing with hospital staff (7.3). Married patients (5.9) worried more about their Sex life than widowed patients (7.5).

Occupational status also appeared to influence how patients ranked categories of need. As predicted, Getting information was less worrying for lower-middle status groups (5.1 blue collar, 5.2 white collar) than for professionals (3.3); however, it was also a more worrying issue for those in home duties (3.5). Retired patients (5.8) were less worried about Work than white-collar (3.8) or skilled workers (4.2) but were more worried about their Social life (3.9). Patients engaged in domestic duties also rated Social life of more concern (4.8) than higher status professions (6.3). Dealing with hospital staff caused more worry for retired patients (5.2) than working class patients (7.1).

**Table II** Mean needs ranking as a function of primary tumour site

| *Primary tumour* | *Family* | *Stress* | *Information* | *Money* | *Work* | *Social* | *Sex* | *Staff* |
|---|---|---|---|---|---|---|---|---|
| Breast (72) | 2.2 | 2.7 | 4.2 | 4.3 | 4.6 | 4.9 | 6.1 | 6.2 |
| Lymphoma (16) | 2.6 | 4.3 | 3.5 | 4.5 | 4.5 | 4.6 | 5.3 | 6.1 |
| Lung (13) | 1.5 | 3.0 | 3.6 | 4.3 | 6.1 | 4.9 | 6.3 | 4.9 |
| Cervical (13) | 1.7 | 2.9 | 4.7 | 2.7 | 4.8 | 5.2 | 6.2 | 6.9 |
| Head/neck (9) | 2.6 | 2.2 | 3.6 | 5.4 | 4.9 | 4.1 | 6.1 | 6.6 |
| Melanoma (7) | 1.2 | 2.6 | 3.4 | 5.4 | 5.3 | 4.6 | 7.0 | 6.0 |
| Others (58) | 1.8 | 2.6 | 3.4 | 5.4 | 5.3 | 4.6 | 7.0 | 6.0 |
| Total (188) | 2.0 | 3.1 | 4.0 | 4.3 | 4.8 | 4.8 | 6.1 | 6.1 |

Difference in mean ranks significant at $P<0.05$. Stress: lymphoma > breast; lymphoma > head/neck; others > breast. Money: head/neck > cervical; lymphoma > cervical. Staff: cervical > lung.

Domestic situation and type of treatment influenced rankings minimally. Living with parents was associated with least worry over Family issues (3.6). Social life caused less worry when children were present (5.3) than when living with a partner only (4.4); conversely, Sex life was more worrying in traditional family situations. Analysis for the effect of treatment type showed greatest concern over Getting information in patients receiving chemotherapy only (3.1) compared with those who also had surgery (5.1). Patients on chemotherapy only were the least worried about Work (6.0) and Sex life (7.5).

### Discussion

Although the data presented are from a heterogeneous group of cancer patients, it is clear that most patients ranked the different categories of psychosocial need in a very similar order of importance, with Family, Dealing with emotional stress, and Getting information assigned the top three priorities (in 96% of the sample). These findings are in accordance with results from previous studies (Stam *et al.*, 1986; Houts *et al.*, 1986; Sullivan *et al.*, 1986). Therefore, psychosocial intervention should focus on these principal areas of need for cancer patients in order to be maximally effective. Innovative interventions should be designed and evaluated to provide psychosocial support for patients in the critical areas of family interaction, effective stress management and access to information.

Analysis of the interactions between relative rankings and demographic variables provided preliminary evidence supporting the validity of this ranking instrument for prioritising eight major areas of psychosocial concern. Our initial hypotheses were confirmed with the exception of age and gender effects on the rankings of Work and Social life. We also found unpredicted effects on Dealing with stress related to marital and occupational status, particularly in the 19% of patients who were widowed or divorced. Despite the positive findings at this stage, the validity of this simple approach to ranking psychosocial need in cancer patients must be viewed with caution. We have undertaken more extensive validation of the instrument by factor analysing an expanded set of items generated using the focus group technique; the results of this analysis will be presented in a future publication.

The sex difference in Dealing with stress is a clear example of the need for a healthy scepticism in drawing implications about intervention at this stage. Male patients reported less worry about Dealing with stress than females. However, this difference could be attributed to a disinclination of males to admit to emotional stress rather than to a true differential in needs and emphasises the fact that allocation of support should not be based solely on self-reported levels of emotional distress. Scales of psychological well-being may prove useful in determining the need for such intervention more accurately. The fact that the order of psychosocial needs did not vary as a function of duration of disease (apart from the modest decrease in ratings for Work) suggests that support in the major areas of need may just as important during follow-up as it is at diagnosis. Again, this interpretation requires follow-up since it is based on cross-sectional data with a highly skewed distribution of time since diagnosis. Priorities of need may change dramatically in patients who are no longer under active treatment.

The choice of very general content areas was deliberate since the alternative of describing specific situations was destined to become exhaustive. In the absence of clear guidelines from our review, we felt it was important to begin at the broadest level identified in the literature and to define the areas empirically by further research. Variation in interpretation of the items has been tested by factor analysis of an expanded set of items generated by the qualitative-research base of focus groups (to be described in a subsequent publication). As one example of the potential for ambiguity, Sex life was generally given a low priority by patients in this study. This is in conflict with the findings of Heinrich *et al.* (1984), who noted that 90% of cancer patients reported difficulties in sexuality. Patients may have interpreted Sex life as referring only to sexual intercourse, or to more general aspects of human relationship including the various forms of physical contact and expressions of affection.

We observed that retired patients and older patients rated Social life more highly than younger groups of patients, an important association which was not among our list of initial predictions. Effective intervention for older patients must take account of the higher value this age-group places on social functioning.

The results in Table II suggest that head and neck cancer patients, and breast cancer patients, suffered more stress than other groups. This could be explained by the visibility of tumours in the head and neck region and the trauma associated with the loss of all or part of a breast. The loss of a breast alters the preception of femininity in breast cancer patients and this may contribute directly to increased distress.

The observation that demographic variables influence the perception and ranking of psychosocial issues in cancer patients argues that intervention or support mechanisms be tailored to the requirements of individual patients. Our data confirmed that Family issues, Dealing with emotional stress, and Getting information were the three major concerns for this population of cancer patients currently undergoing treatment. It is now critically important to obtain feedback from individual patients on how these specific psychosocial needs are perceived in order to understand and meet them. Of course, not every patient who reveals concerns about psychosocial aspects of the disease will need or want our support or intervention. For example, any attempt to confront the avoidance and denial which typically pervade family dynamics will be met with profound hostility and we must have exceptionally effective alternatives to replace these potent defences. Nevertheless, increased knowledge of psychosocial needs and coping difficulties would allow the precise targeting of psychosocial interventions in an attempt to improve the quality of life of patients with cancer.

This work was supported by a NSW State Cancer Council Patient Care Research Award (1988-9). We are grateful to Prof. Allan Langlands and the staff and patients of the Department of Radiation Oncology, Westmead Hospital.

### References

AARONSON, N.K., BULLINGER, M. & AHMEDZAI, S. (1988). A modular approach to quality of life assessment in cancer clinical trials. *Rec. Results Cancer Res.*, **111**, 231.

CLARK, A. & FALLOWFIELD, L.J. (1986). Quality of life measurements in patients with malignant disease: a review. *J. R. Soc. Med.*, **79**, 165.

DEPARTMENT OF HEALTH, NSW (1986). *An Epidemiological Profile of the Western Metropolitan Health Region*. Planning and Research Unit, Western Metropolitan Health Region.

DEROGATIS, L.R. (1986). Psychology in cancer medicine: a perspective and overview. *J. Consult. Clin. Psychol.*, **54**, 632.

DEROGATIS, L.R. & SPENCER, P.M. (1986). *Stress, Appraisal and Coping*. Springer: New York.

GOLDBERG, R.J. & WOOL, M.S. (1985). Psychotherapy for the spouses of lung cancer patients: assessment of an intervention. *Psychother. Psychosom.*, **43**, 141.

HEINRICH, R.L., SCHAG, C.C. & GANZ, P.A. (1984). Living with cancer: the cancer inventory of problem situations. *J. Clin. Psychol.*, **40**, 972.

HOUTS, P.S., YASKO, J.M., KAHN, S.B., SCHELGEL, G.W. & MARCONI, K.M. (1986). Unmet psychological, social, and economic needs of persons with cancer in Pennsylvania. *Cancer*, **58**, 2355.

JACOBS, C., ROSS, R.D., WALKER, I.M. & STOCKDALE, F.E. (1983). Behavior of cancer patients: a randomized study of the effects of education and peer support group. *Am. J. Clin. Oncol. Cancer Clin. Trials*, **6**, 347.

NOLAN, T., ZVALGULIS, I. & PLESS, B. (1987). Controlled trial of social work in childhood chronic illness. *Lancet*, **ii**, 411.

SELBY, P. & ROBERTSON, B. (1987). Measurement of quality of life in patients with cancer. *Cancer Surv.*, **6**, 521.

STAM, H.J., BULTZ, B.D. & PITTMAN, C.A. (1986). Psychosocial problems and interventions in a referred sample of cancer patients. *Psychosom. Med.*, **48**, 539.

SULLIVAN, A.L., DEVINE, R.J., BOWEN-THOMAS, J.E. & TATTERSALL, M.H.N. (1986). Cancer patients' needs. *Cancer Forum*, **10**, 92.

WATSON, M. (1983). Psychosocial intervention with cancer patients: a review. *Psychol. Med.*, **13**, 839.